# Genome-wide analysis of miRNAs in *Carya cathayensis*

**DOI:** 10.1186/s12870-017-1180-6

**Published:** 2017-11-29

**Authors:** Zhi-chao Sun, Liang-sheng Zhang, Zheng-jia Wang

**Affiliations:** 1 State Key Laboratory of Subtropical Silviculture, School of Forestry and Biotechnology, Zhejiang Agriculture and Forestry University, Hangzhou, 311300 China; 2 Center for Genomics and Biotechnology; Fujian Provincial Key Laboratory of Haixia Applied Plant Systems Biology; Ministry of Education Key Laboratory of Genetics, Breeding and Multiple Utilization of Corps; State Key Laboratory of Ecological Pest Control for Fujian and Taiwan Crops; College of Life Science, Fujian Agriculture and Forestry University, Fuzhou, 350002 China; 3 School of Forestry and Biotechnology, Zhejiang A and F University, Dong Hu Campus, 88 Northern Circle Road, Linan, 311300 China

**Keywords:** Hickory, Female flower, miRNA, Phylogenetic, Quantitative real-time PCR

## Abstract

**Background:**

MicroRNA (miRNA) plays an important role in plant development regulation. Hickory is an economically important plant in which the amount of flowering determines its production.

**Results:**

Here, 51 conserved miRNAs, which belong to 16 families and 195 novel miRNAs were identified in hickory genome. For each conserved miRNA family, we used sequences from hickory and other plants to construct a phylogenetic tree, which shows that each family has members in hickory. Some of the conserved miRNA families (i.e., miR167 and miR397) have more members in hickory than in other plants because of gene expansion. MiR166 exhibited tandem duplication with three copies being observed. Many members of these conserved miRNA families were detected in hickory flowers, and the expression patterns of target genes were opposite to those of the related miRNAs, indicating that miRNAs may have important functions in floral regulation of hickory.

**Conclusions:**

Taken together, a comprehensive analysis was conducted to identify miRNAs produced in hickory flower organs, demonstrating functional conservation and diversity of miRNA families among hickory, *Arabidopsis*, grape, and poplar.

**Electronic supplementary material:**

The online version of this article (doi: 10.1186/s12870-017-1180-6) contains supplementary material, which is available to authorized users.

## Background

MicroRNAs (miRNAs) are classified as small non-coding RNA molecules, whose length range from 18 to 24 nucleotides, acting as central regulators of gene expression [1]. They negatively regulate gene expression by complementarily binding to the open reading frames (ORFs) or untranslated regions (UTRs) of target messenger RNAs. Mature miRNAs are derived from primary miRNAs transcribed from specific MIR genes. These precursor molecules are cleaved by the Dicer-Like1 (*DCL1*) protein, forming a miRNA/miRNA* complex, which is divided into miRNA and miRNA* after transporting to the cytoplasm [2]. MiRNAs are bounded by Argonaute proteins, forming a part of the RNA-induced silencing complex (RISC). RISC then interacts with the target mRNAs at the paired region, cleaving the RNA of target genes [3]. Plant development (i.e., flower development) and its response to environmental stresses are regulated by MiRNAs [4–7]. Studies have shown that miR156 and miR172 families play important roles in vegetative phase changes in *Arabidopsis* [8]. Additionally, the miR159, miR319 and miR390 families play roles in controlling the flowering time [9–11].

In 1993, the first miRNA (lin-4) was discovered and identified in *Caenorhabditis elegans* [12]. In 2001, many miRNAs were identified in a number of animal species via cloning and sequencing [13], and many additional miRNA were revealed by bioinformatics prediction and cloning. MiRNAs can be easily identified based on sequences. However, it is difficult to detect species-specific miRNAs. The cloning method is laborious and can only be used to identify miRNAs on a small scale. Alternatively, high-throughput sequencing is a powerful approach for identifying miRNAs [14]. High-throughput sequencing technology can yield abundant reads and detect the expression of minimally abundant small RNAs (sRNAs). This methodology has become the technique of choice for sequencing genomes, transcriptomes and small RNA transcriptomes [15]. The expression of sRNAs at different developmental stages can be profiled by high-throughput sequencing [16].

The evolution and function of miRNAs are revealed by miRNA-based studies from a phylogenetic perspective. Evolutionary conservation enables the identification of miRNAs for which homologs have previously been reported in other species [17]. miRNA genes are frequently expressed in a temporally and spatially regulated manner. Also, they play a fine-tuning role, by which the expressions of protein-coding genes are regulated. miRNA sequences are highly conserved among different plants as well as their functions [9, 10]. In angiosperms, functional identification of miRNAs is less available, so analysis of the miRNA repertoire is necessary to trace out the miRNA targets and the functional diversification in land plants.

Hickory (*Carya cathayensis*), an important economic woody plant, is mainly distributed in Zhejiang and Anhui provinces of China. Hickory has a long juvenile phase, and females and males present different flowering times, imposing a constraint on breeding and stable annual production. The morphological turning point from vegetative to reproductive stage appears in late March, when male floral buds dehisce and pollen releases. Huang et al., (2007) have observed five distinct stages for hickory female floral development including the floral undifferentiated stage, flower bud differentiation and the stage of formation and development of the inflorescence, of the female involucre, and of the pistil [18–20].

Thus, it is necessary to identify miRNAs and analyse sequence divergence of miRNAs in hickory, especially their relation to flower development. In this study, hickory miRNAs were investigated by RNA-seq technology, and those related to March Flower (MF) (floral undifferentiated stage) and April Flower (AF) (flower bud differentiation) were further analysed. Many miRNAs were obtained and compared with homologous sequences from the genomes of other plants. A comprehensive phylogenetic analysis were also conducted for hickory miRNA family members and their homologs from three other land plant species.

## Results

### Identification and expression analysis of conserved and novel miRNAs in hickory

To identify conserved miRNAs associated with development of female flower, miRNAs were predicted using hickory genome (unpublished) with SOAP software, and their secondary structures were also predicted. 51 conserved mature miRNAs of 16 families and their precursor sequences were identified in hickory genome. The minimal folding energy (MFE) of the precursor sequences ranged from −26.1 to −99.62 kcal.mol^−1^, with a mean of −56.27 kcal.mol^−1^. The largest family was the miR167 family consisting of six members, followed by miR156 family, which included five members (Table 1). The expression of miRNAs was calculated with RPKM (Reads Per Kilobase of exon model per Million mapped reads) [21]. The expression of these predicted conserved miRNAs varied from 0.30 to 39,508.53, with means of 832.42 (MF) and 1304.09 (AF) (Additional file 1: Table S4). Among these families, the miR166 family was the most abundantly expressed, while the miR167 family exhibited the second most abundant expression. Thus, miR166 and miR167 may play important roles in flower development of hickory. Several miRNA families (miR160, miR390 and miR168 families) were moderately expressed (Additional file 1: Table S4), whereas only a few read counts were detected for some miRNA families. Thirty miRNAs exhibited uridine at the 5′ end, accounting for 58.82% of all conserved miRNAs, whereas adenine, guanine and cytosine were the first bases in 8 (15.69%), 8 (15.69%), and 5 (9.80%) miRNAs, respectively (Additional file 2: Figure S1a). The presence of uridine as the first base is characteristic of miRNAs, this region interacts with *AGO1*, together with the miRNA/RISC complex [22–24]. We observed a bias toward adenine and guanine at the 5′ end, which coincided with previous studies in flowers [25].

**Table 1:** The conserved miRNA family in *Carya cathayensis* and other plant

Names	miR156	miR160	miR162	miR166	miR167	miR168	miR169	miR171	miR172	miR319	miR390	miR395	miR397	miR398	miR408	miR482
*Ath*	10	3	2	7	4	2	14	3	5	3	2	6	2	3	1	0
*Ptc*	12	8	2	17	8	2	33	13	9	9	4	11	3	3	1	4
*Vvi*	9	5	1	8	5	1	25	10	4	5	1	14	1	3	1	1
*Cca*	5	2	2	3	6	1	3	4	3	1	2	1	3	1	1	1

*Ath Arabidopsis thaliana*, *Ptc Populus trichocarpa, Vvi Vitis vinifera*, *Cca Carya cathayensis*

In total, 195 predicted novel mature miRNAs and precursor sequences were identified from different floral developmental stages in hickory (Additional file 1: Table S4). The average minimal folding energy of the precursor sequences was −54.57 kcal.mol^−1^, ranging from −18.7 to −128.5 kcal.mol^−1^ (Additional file 1: Table S4). Uridine was the first nucleotide in nearly half of the novel mature miRNA sequences, which was consistent with results in soybean (Additional file 2: Figure S1b). The expression abundant of novel miRNAs varied from 0.28 to 5825.56, with means of 44.77 (MF) and 122.78 (AF), which was much lower than the conserved miRNAs. Some novel miRNA sequences with tissue-specific expression, including cca-miR27, cca-miR36, cca-miR48, cca-miR113 and cca-miR176, were only detected in the AF library, while cca-miR127 was only expressed in the MF library. Some miRNAs, such as cca-miR87, cca-miR105 and cca-miR122, presented higher expression in the AF library than that in the MF library (Additional file 1: Table S4). These results suggested that these novel miRNAs may play important roles in the development at the flowering stage.

We compared the miRNA expression levels between MF and AF. miRNAs that exhibited AF/MF ratios greater than 2 were considered to indicate differential expression. The expression of twenty-two conserved and twenty-six novel miRNA sequences were different, and most of these miRNAs were up-regulated in AF (Additional file 1: Table S5). In addition, some miRNAs (cca-miR27, cca-miR113, cca-miR176, and miR156j) were only detected in the AF library (Additional file 1: Table S5), showing that certain miRNAs are tissue-specific [26]. No large changes in miRNA expression (expression ratios greater than 10.00) were observed, except for miR160a, which presented a ratio of 10.43. The miRNAs exhibited high expression levels in both AF and MF (expression values of 355.75 and 34.12, respectively). The conserved miR156, miR166, miR169 and miR171 families were differentially up-regulated in AF, which was consistent with the roles they play in floral organ development (Additional file 2: Figure S2, Additional file 1: Table S5). New species-specific miRNAs were considered novel miRNAs if they had evolved recently and were frequently expressed at low levels compared with conserved miRNAs as reported in *Arabidopsis* and wheat. Conversely, cca-miR27, cca-miR65, cca-miR113, cca-miR164 and cca-miR176 exhibited expression values greater than 100 in the AF libraries (Additional file 1: Table S5).

We used quantitative real-time PCR (qRT-PCR) to further analyze the expression of 15 conserved miRNAs and 12 novel miRNA candidates in different tissues and also different female floral development stages (Additional file 2: Figure S2). Both qRT-PCR and Solexa sequencing demonstrated that the conserved miRNA sequences as well as the novel miRNAs were up-regulated during flower development, except for miR393a and miR408 (Additional file 2: Figure S2, Additional file 1: Table S5). Solexa sequencing indicated that cca-miR27 and cca-miR113 were only detectable at the AF floral stage, whereas qRT-PCR indicated that they were expressed at both floral stages (Additional file 2: Figure S2); this disparity likely occurred because deep sequencing could not detect the entire real distribution of these miRNAs, and some miRNAs were therefore not detected. The expression of most miRNAs was ubiquitous in all studied tissues, suggesting that they might have multiple functions in plant growth and development. Some miRNAs (cca-miR158, miR166b-3p, miR171a-3p, and miR319d) were highly expressed in flowers, while nearly all miRNAs (e.g., cca-miR27, cca-miR67, miR398a, miR408) were highly expressed in leaves. Many miRNAs, including cca-miR129, cca-miR140, cca-miR158, miR169, miR393, miR398, and miR408, were hardly detectable in roots, leaves and fruit (Additional file 2: Figure S3).

### Phylogenetic analysis of conserved miRNAs in hickory

**Fig. 1:**
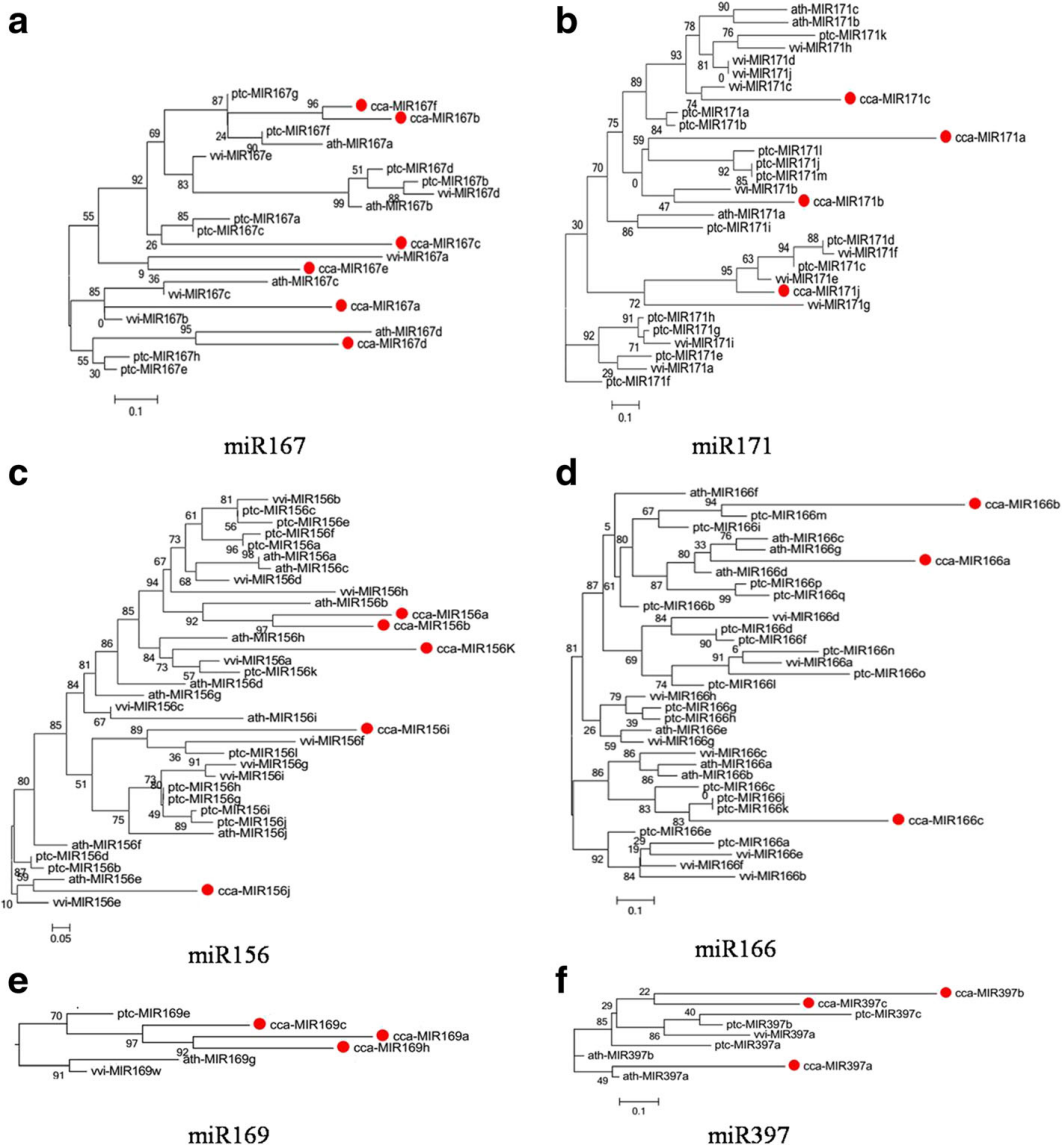
The maximum likelihood (ML) phylogenetic tree reconstruction using precursor miRNA family sequences from Arabidopsis (ath), grape (vvi), popar (ptc), and hickory (cca). MUSCLE alignment and ML were used for tree generation. The phylogenetic tree for miR167 (**a**), miR171 (**b**), miR156 (**c**), miR166 (**d**), miR169 (**e**), miR397 (**f**). The miRBase accession numbers as follows: ath-MIR167a (MI0000208), ath-MIR167b (MI0000209), ath-MIR167c (MI0001088), ath-MIR167d (MI0000975), ptc-MIR167a (MI0002235), ptc-MIR167b (MI0002236), ptc-MIR167c (MI0002237), ptc-MIR167d (MI0002238), ptc-MIR167e (MI0002239), ptc-MIR167f (MI0002240), ptc-MIR167g (MI0002241), ptc-MIR167h (MI0002242), vvi-MIR167a (MI0006515), vvi-MIR167b (MI0006516), vvi-MIR167c (MI0006517), vvi-MIR167d (MI0006518), vvi-MIR167e (MI0006519), ath-MIR171a (MI0000214), ath-MIR171b (MI0000989), ath-MIR171c (MI0000990), ptc-MIR171a (MI0002277), ptc-MIR171b (MI0002278), ptc-MIR171c (MI0002279), ptc-MIR171d (MI0002280), ptc-MIR171e (MI0002281), ptc-MIR171f (MI0002282), ptc-MIR171g (MI0002283), ptc-MIR171h (MI0002284), ptc-MIR171i (MI0002285), ptc-MIR171j (MI0007034), ptc-MIR171k (MI0005114), ptc-MIR171l (MI0007032), ptc-MIR171m (MI0007033), vvi-MIR171a (MI0006536), vvi-MIR171b (MI0006537), vvi-MIR171c (MI0006538), vvi-MIR171d (MI0006539), vvi-MIR171e (MI0006540), vvi-MIR171f (MI0006541), vvi-MIR171g (MI0007950), vvi-MIR171h (MI0006542), vvi-MIR171i (MI0006543), vvi-MIR171j (MI0031743), ptc-MIR156a (MI0002184), ptc-MIR156b (MI0002185), ptc-MIR156c (MI0002186), ptc-MIR156d (MI0002187), ptc-MIR156e (MI0002188), ptc-MIR156f (MI0002189), ptc-MIR156g (MI0002190), ptc-MIR156h (MI0002191), ptc-MIR156i (MI0002192), ptc-MIR156j (MI0002193), ptc-MIR156k (MI0002194), ptc-MIR156l (MI0022040), ath-MIR156a (MI0000178), ath-MIR156b (MI0000179), ath-MIR156c (MI0000180), ath-MIR156d (MI0000181), ath-MIR156e (MI0000182), ath-MIR156f (MI0000183), ath-MIR156g (MI0001082), ath-MIR156h (MI0001083), ath-MIR156i (MI0019232), ath-MIR156j (MI0019234), vvi-MIR156a (MI0006485), vvi-MIR156b (MI0006486), vvi-MIR156c (MI0006487), vvi-MIR156d (MI0006488), vvi-MIR156e (MI0006489), vvi-MIR156f (MI0006490), vvi-MIR156g (MI0006491), vvi-MIR156h (MI0007939), vvi-MIR156i (MI0006492), ptc-MIR166o (MI0002232), ptc-MIR166p (MI0002233), ptc-MIR166q (MI0002234), vvi-MIR166a (MI0006507), vvi-MIR166b (MI0006508), vvi-MIR166c (MI0006509), vvi-MIR166d (MI0006510), vvi-MIR166e (MI0006511), vvi-MIR166f (MI0006512), vvi-MIR166g (MI0006513), vvi-MIR166h (MI0006514), ath-MIR169a (MI0000212), ath-MIR169b (MI0000976), ath-MIR169c (MI0000977), ath-MIR169d (MI0000978), ath-MIR169e (MI0000979), ath-MIR169f (MI0000980), ath-MIR169g (MI0000981), ath-MIR169h (MI0000982), ath-MIR169i (MI0000983), ath-MIR169j (MI0000984), ath-MIR169k (MI0000985), ath-MIR169l (MI0000986), ath-MIR169m (MI0000987), ath-MIR169n (MI0000988), ptc-MIR169a (MI0002245), ptc-MIR169b (MI0002252), ptc-MIR169c (MI0002253), ptc-MIR169d (MI0002254), ptc-MIR169e (MI0002255), ptc-MIR169f (MI0002256), ptc-MIR169g (MI0002257), ptc-MIR169h (MI0002258), ptc-MIR169i (MI0002259), ptc-MIR169j (MI0002260), ptc-MIR169k (MI0002261), ptc-MIR169l (MI0002262), ptc-MIR169m (MI0002263), ptc-MIR169n (MI0002264), ptc-MIR169o (MI0002265), ptc-MIR169p (MI0002266), ptc-MIR169q (MI0002267), ptc-MIR169r (MI0002268), ptc-MIR169s (MI0002269), ptc-MIR169t (MI0002270), ptc-MIR169u (MI0002271), ptc-MIR169v (MI0002272), ptc-MIR169w (MI0002273), ptc-MIR169x (MI0002274), ptc-MIR169y (MI0002275), ptc-MIR169z (MI0002276), ptc-MIR169aa (MI0002246), ptc-MIR169ab (MI0002247), ptc-MIR169ac (MI0002248), ptc-MIR169ad (MI0002249), ptc-MIR169ae (MI0002250), ptc-MIR169af (MI0002251), ptc-MIR169ag (MI0022041), vvi-MIR169a (MI0006521), vvi-MIR169b (MI0007940), vvi-MIR169c (MI0006523), vvi-MIR169d (MI0006524), vvi-MIR169e (MI0006525), vvi-MIR169f (MI0006526), vvi-MIR169g (MI0006527), vvi-MIR169h (MI0007941), vvi-MIR169i (MI0007942), vvi-MIR169j (MI0006528), vvi-MIR169k (MI0006529), vvi-MIR169l (MI0007943), vvi-MIR169m (MI0006530), vvi-MIR169n (MI0007944), vvi-MIR169o (MI0007945), vvi-MIR169p (MI0006531), vvi-MIR169q (MI0007946), vvi-MIR169r (MI0006532), vvi-MIR169s (MI0006533), vvi-MIR169t (MI0006534), vvi-MIR169u (MI0006535), vvi-MIR169v (MI0007947), vvi-MIR169w (MI0007948), vvi-MIR169x (MI0007949), vvi-MIR169y (MI0006522), ath-MIR397a (MI0001015), ath-MIR397b (MI0001016), ptc-MIR397a (MI0002332), ptc-MIR397b (MI0002333), ptc-MIR397c (MI0002334), vvi-MIR397a (MI0007956)

**Fig. 2:**
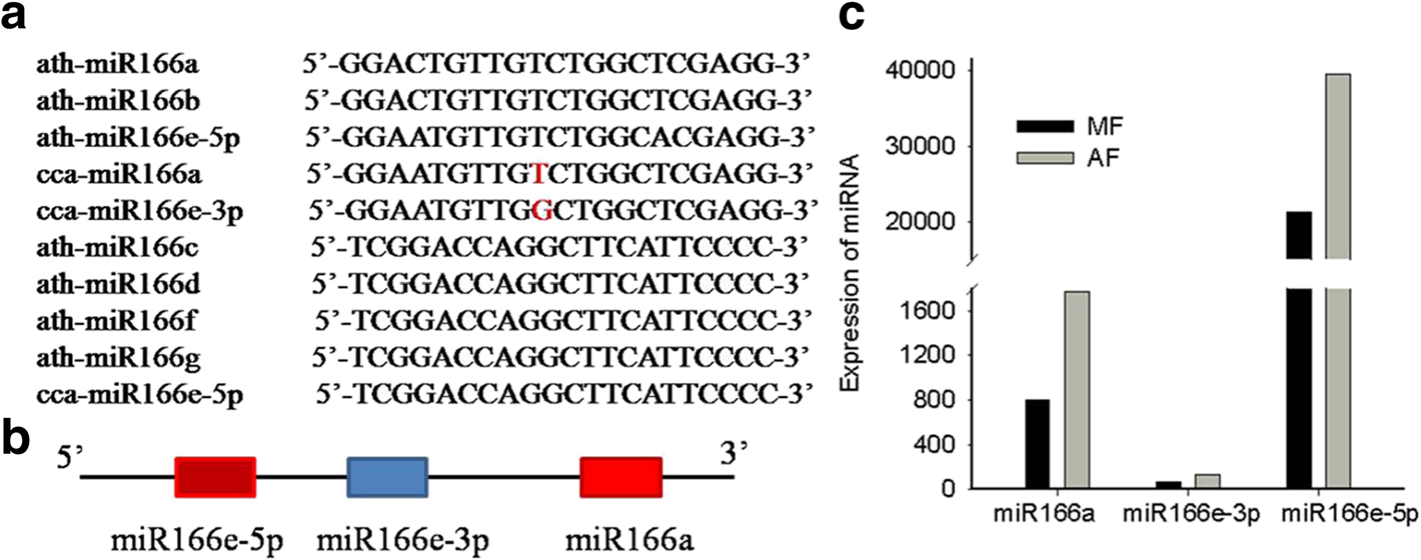
Tandem duplication in the evolution of the miR166 family. **a** Alignment of mature miR166 of hickory with Arabidopsis. **b** Showing tandem duplication event of miR166e-5p and miR166a. **c** Expression of miR166a, miR166e-5p, and miR166e-3p

miRNA precursors were retrieved from miRBase21.0 for three species (*Arabidopsis*, grape, poplar) (Additional file 2: Figure S1), and a phylogenetic analysis of the miRNA precursor sequences was conducted. The obtained maximum likelihood (ML) tree (Fig. 1; Additional file 2: Figure S4) indicated that the data could be divided into three categories. Some hickory miRNAs were present in every clade (miR156, miR162, miR167, miR168, miR390, miR397, miR408 and miR482), whereas other hickory miRNAs were observed in most clades (miR166, miR171, miR172 and miR398), and some hickory miRNAs were included in only one clade (miR160, miR169, miR319 and miR395). Among the first group, there were four miR167 (miR167a-d) in *Arabidopsis*, five (miR167a-e) in grape, eight (miR167a-h) in poplar and six in hickory. The phylogenetic results showed that miR167 was partitioned into four major clades and that hickory miR167 was included in every clade (Fig. 1a). For miR156 and miR397, hickory miRNAs were observed in every clade in the phylogenetic tree (Fig. 1c, f). Among the second group, the phylogenetic results showed that miR171 was partitioned into four major clades. Only one clade did not contain hickory miR171 (Fig. 1b). Among the third group, for miR169, hickory miRNA was found in only one clade of the phylogenetic tree, and three members were found in hickory, while only one miRNA was observed in the other species (Fig. 1e). miR166 exhibited tandem duplication (miR166a and miR166e-3p), with the two miRNAs showing only a one-nucleotide difference but distinct differences in expression (Fig. 2). This tandem arrangement provides a simple explanation for the existence of different functions.

### Sequence conservation and divergence of conserved miRNAs in eudicots

**Fig. 3:**
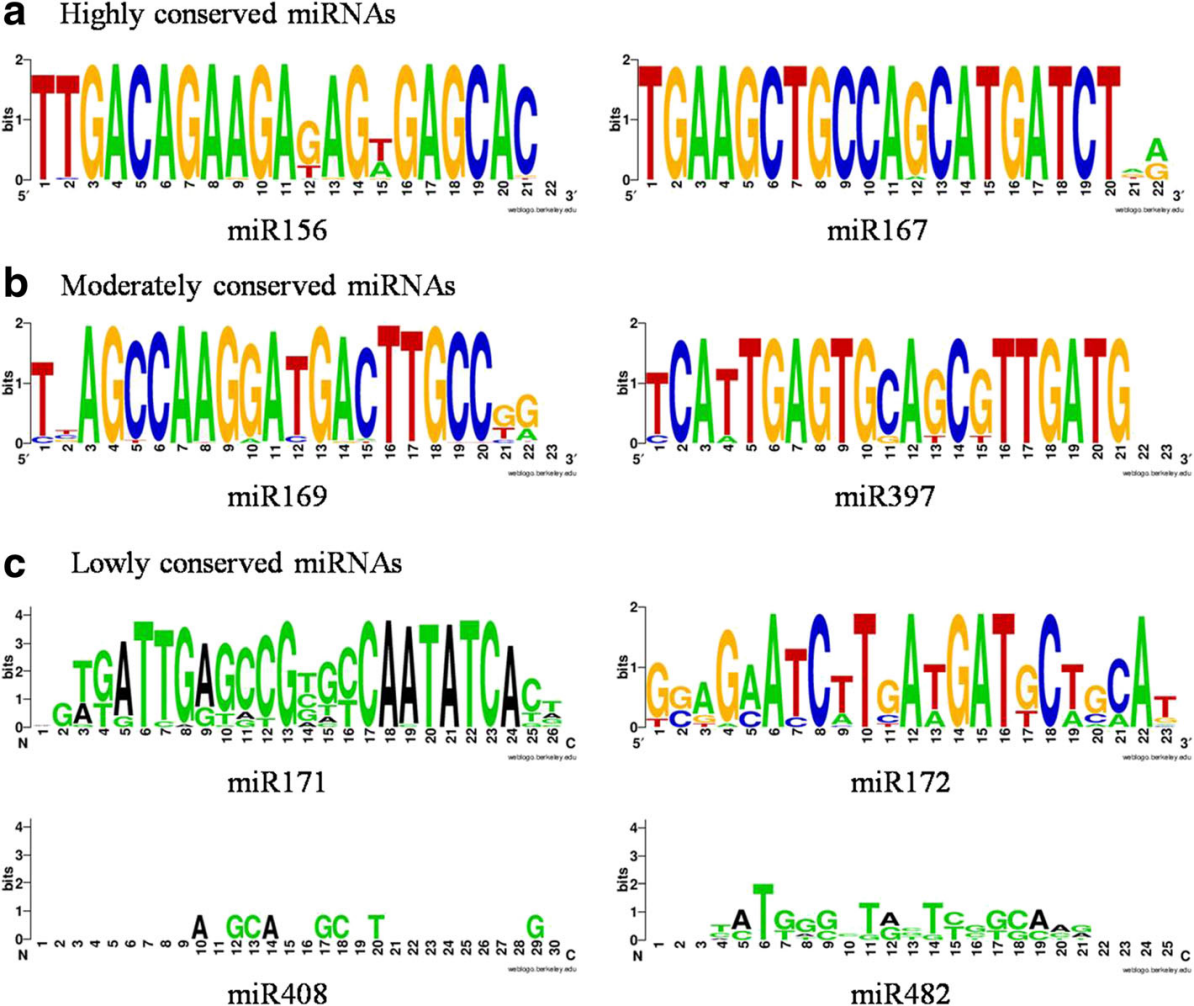
Sequence logo showing a consensus sequence generated from the multiple alignments of miRNA families from four different plant species. **a** The miR156 and miR167 represent highly conserved miRNAs. **b** The miR169 and miR397 represent moderately conserved miRNAs. **c** The miR171, miR172, miR408, and miR482 represent lowly conserved miRNAs

Orthologous and paralogous sequences in the gene structures of miRNA family members in the eudicots *Arabidopsis*, grape, poplar and hickory were compared (Additional file 2: Figure S2). The sequence alignment of mature miRNAs revealed a conserved consensus, with few variations. We further analyzed the motifs of the conserved miRNAs through a WebLogo analysis. The level and number of degenerate positions indicated by multiple alignments revealed degrees of conservation between the sequences (Fig. 3 and Additional file 2: Figure S5). Notably, nine miRNA families showed highly conserved sequences with 0–3 nucleotide substitutions in homologous regions. The highly conserved sequences were miR160, miR162, miR166, miR167, miR168, miR319, miR390, miR398 and miR408. These highly conserved miRNAs may have the same target mRNAs. The remaining sequences showed variable divergence, as indicated by different target gene products of multiple alignments of sequences. The degree of degeneracy in a miRNA sequence and the number of targets may be correlated. miR169, miR171 and miR482 were assembled according to various strategies and exhibited many variable positions (Fig. 3). However, the miR482 family contained the greatest number of nucleotide substitutions and was observed in woody plants (Fig. 3).

### Target prediction and analysis of expression patterns

Based on the investigation of miRNA functions, 10 conserved and 2 novel differentially expressed miRNAs were selected as candidate miRNAs related to flower development (Table 2). In most of the miRNA families, including miR156, miR166, miR167, miR169 and miR171, some targets were involved in male and female flower development. These miRNAs were differentially expressed during flower development, except for miR168, miR319 and miR390 (Additional file 1: Table S5).

**Table 2:** Information of plant miRNAs and their targets in *Carya cathayensis*

miRNA	Function	Targets	Reference	Target	Annotation
cca-miR27				contig06943	
cca-miR38				contig23321	*LOM1*
				contig24104	*LOM3*
miR156	Juvenile-to-adult transition	Squamosa promoter-binding protein, putative (*SBP*)	[43]	contig03115	*SPL9*
				contig20796	*SPL10*
miR160	Flower organs development	Auxin response factors (*ARF*)	[44]		
miR166	Leaf polarity; shoot apical meristem and lateral organ formation	Class III homeodomain leucine zipper protein (*HD-ZIP III*)	[27, 28]	contig26884	HD-ZIP III
miR167	Reproductive development, fertility of anthers	*ARF*	[32]		
miR168	miRNA pathway regulation	Argonaute 1(*AGO1*)	[45]	contig19324	*AGO1*
miR169	Juvenile-to-adult transition	Nuclear Factor Y, Subunit A1 (*NF-YA 1*)	[46]	contig23375	*NF-YA1*
miR171	Flower development	Lost Meristems (*LOM*)	[30]	contig04244	*LOM1*
				contig09385	*LOM3*
				contig23998	*LOM3*
miR172	Juvenile-to-adult transition	Apetala2 (*AP2*)	[1]	contig05419	*TOE2*
				contig05202	*TOE1*
miR319	Flower development	TCP Domain Protein(*TCP*)	[47]		
miR390	Developmental timing and patterning	*TAS3* transcripts	[48]		

**Fig. 4:**
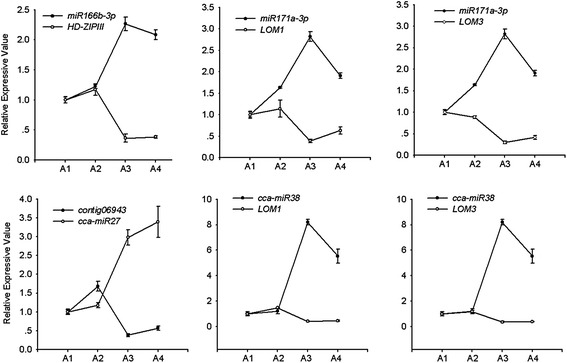
RT-PCR analysis of miRNA and its targets expression. RT-PCR was performed with different flower developmental stages. Each bar represents an average of three independent reactions, including both biological and technical replicates. Error bars indicate SD

A class III homeodomain-leucine zipper (*HD-ZIPIII*) was previously described and validated as a miR166 target in *Arabidopsis* [27, 28]. Therefore, we analyzed its expression at different times during flower development and in different tissues. The qRT-PCR analysis indicated accumulation of *HD-ZIPIII* transcripts in early flowers (A2), with similar accumulation patterns in the leaves and stems (Fig. 4 and Additional file 2: Figure S6). The expression of *HD-ZIPIII* decreased in mature hickory flowers (A3), and reduced accumulation was observed in the leaves and flowers, indicating that *HD-ZIPIII* is strongly down-regulated along with the flower development in hickory. This observation is in agreement with the functional role of this transcription factor in regulating hickory flower development [29]. *HD-ZIPIII* expression is inversely correlated with the expression pattern of miR166 (Fig. 4), suggesting a regulatory role of this miRNA associated with *HD-ZIPIII* in hickory plants during flower development.

miR171 has been reported to down-regulate lost meristem (*LOMs*) genes during phase transition in *Arabidopsis* [30]. The miR171-targeted *LOM* genes, which encode GRAS family members known to maintain meristem cell polarity, are involved in the regulation of *SPL* activity [30]. In hickory, the transcripts of *LOM* genes preferentially accumulated in early flowers (A2), which showed higher expression levels than mature flowers (A3) (Fig. 4), whereas *LOM* gene expression was markedly decreased in mature flowers (A3) (Fig. 4). This expression pattern was inversely correlated with up-regulation of miR171 in the same developmental stages (Fig. 4), suggesting that miR171 regulates *LOM* genes during flower development in hickory.

Expression analysis indicated that miRNA targets exhibited distinct expression patterns during flower development. We also determined that several miRNAs regulated their targets in the same manner. For example, the targets of cca-miR27, cca-miR38, miR166b and miR171a were all down-regulated to different extents (A2-A3) in the flowering stage (Fig. 4), showing opposite expression patterns to the related miRNAs, indicating that the cca-miR27, cca-miR38, miR166b and miR171a families negatively regulate their targets.

## Discussion

In recent years, a large number of conserved miRNAs and species-specific miRNAs have been identified using high-throughput sequencing [5]. This method presents a high-throughput capacity, allowing large-scale detection of miRNAs and high-sensitivity detection of minimally expressed miRNAs [5, 31, 32]. Here, we identified 51 conserved miRNAs belonging to 16 families and 195 novel miRNAs (Additional file 1: Table S4). Through miRNA-seq, we identified more miRNA copies than have been found in other sequenced species, with several copies being observed in some families. Furthermore, certain miRNAs exhibited gene expansion in hickory. Previous studies have indicated that the morphological turning point between vegetative and reproductive stages of hickory emerges during late March, when male floral buds are dehiscent [19]. Expression analysis suggested that some miRNAs exhibited higher expression in mature flowers (A3) compared with early flowers (A2), and the expression patterns of their target genes were in contrast to those of the miRNAs. Analysis of the *Arabidopsis* floral transcriptome indicated that many genes encoding transcription factors were preferentially expressed during early flower development compared with other stages [29]. These early-expressed genes have functions that are associated with reproductive development, suggesting that early flower development requires many more abundantly expressed genes than in mature flowers [29]. Overall, our findings suggest that these miRNAs may be related to flower development.

We analyzed the hickory miRNA families and compared their members with representatives from *Arabidopsis*, grape and poplar. Most of the mature miRNA sequences exhibited high sequence identity and conservation (Fig. 4, Additional file 2: Figure S4), but all of the mature sequences presented some degree of divergence. Multiple alignments enabled the identification of nine highly conserved miRNA families (miR160, miR162, miR166, miR167, miR168, miR319, miR390, miR398 and miR408) and four moderately conserved miRNA families (miR156, miR172, miR395 and miR397). miR169, miR171 and miR482 were belonged to miRNA families with low conservation (Fig. 3, Additional file 2: Figure S4).

The phylogenetic analysis of precursors of different miRNA families from the selected species showed a distinct clustering pattern. The phylogenetic tree for the miRNAs suggested that although the miRNA families were conserved among the four plant species, some miRNA members exhibited new functions because of additional mutation events in the mature miRNA region of the miRNA genes. Overall, these analyses revealed a combination of ancestral relationships and recent lineage-specific diversification. In the phylogenetic analysis, the ML trees were classified into three types. Hickory miR156 and miR167 family members were observed in every clade and belonged to the moderately and highly conserved miRNA groups, respectively (Fig. 1). However, mature miR156s, exhibiting little sequence diversification and differentiated expression, are expected to play a different functional role in hickory flower development. These conserved miRNAs and their conserved target transcription factors highlight the versatile functions of miRNAs and provide further evidence of the phylogenetic distribution of miRNA families, regardless of species boundaries. Additionally, both mature miR156 and miR167, which showed minor sequence variations, can still target similar genes (Fig. 3; Table 1). Thus, the functions of miR157 and miR167 are expected to be conserved among the four species.

Tandem duplication events in miRNA genes result in the formation of paralogous miRNA gene copies located in close proximity to each other on the same chromosome, thus forming miRNA clusters. It was recently demonstrated that miRNAs have been expanded through tandem duplication in the *Arabidopsis*, poplar and grape genomes, with 248 miRNAs belonging to 51 miRNA families being demonstrated to have originated via tandem duplication, including the miR166 family [33]. The clustering of cca-miR166a and cca-miR166e suggests that they originated from recent duplication event, without major sequence changes. The expression of these two mature miRNAs exhibits significant variation (Fig. 2). Some evidence shows that the miR166 family has undergone more intensive diversification through multiple duplication and expansion events since separation from an ancestor. miR166 regulates shoot apical meristem and flower development in *Arabidopsis* by targeting the transcription of *HD-ZipIII* transcription factors [34]. Over-expression of miR166 may destroy the flower structure and could result in a decreased number of pistils and carpels [28]. Pistillate flowers are naked without a perianth in hickory. Regarding biological characteristics, the pistillate flowers of hickory are initiated from a terminal bud that grows on short pod-branches in young hickory trees and persists until reproductive age. Thus, the miR166 family may regulate the pistillate flower structure and floral meristem determination in hickory.

## Conclusions

We conducted a comprehensive analysis of hickory miRNAs produced in flower organs and computationally predicted their putative targets. We further performed bioinformatics analyses of 16 families of conserved miRNAs selected from the most conserved families, according to the biological processes in which they are involved. Our observations demonstrated the functional conservation and diversity of miRNA families among hickory, *Arabidopsis*, grape and poplar. This study provides data on the functional diversification and conservation of miRNA genes and constitutes a basis for further experimental studies of miRNA function in hickory. Some targets of hickory miRNAs have counterparts that have been previously identified and validated in other species, such as *A. thaliana*, *V. vinifera* and *O. sativa* [35–37]. Several known targets of specific miRNAs (primarily transcription factors) control various physiological processes and genetic programs associated with plant metabolism, flowering, hormone signaling and stress responses, as reflected by our data. This dataset is useful for identifying functional miRNA genes involved in hickory flower development and provides clues regarding miRNA evolution in various species.

## Methods

### Materials

Five 15-year-old clones of hickory trees planted at Lin’an, China were used as plant materials. Healthy plants were selected, and samples of the roots, stems, leaves, female and fruits were collected. Apart from different sample tissue collection, the various stages of female floral development were also collected including A1 stage (floral undifferentiated stage 1), A2 stage (floral undifferentiated stage 2), A3 stage (flower bud differentiation), and A4 stage (flower bud formation).

### Conserved and novel miRNAs in hickory

The Raw data were generated from MF and AF sRNA libraries of female flowers [5]. Clean RNAs were mapped to hickory genome (unpublished) using SOAP software. The sRNAs with a perfect match were further analyzed. Sequences matching noncoding RNAs including rRNAs, scRNAs, snoRNAs, snRNAs, and tRNAs were annotated by comparing with sequences in the NCBI GenBank (http://www.ncbi.nlm.nih.gov/blast/Blast.cgi) and Rfam databases (http://www.sanger.ac.uk/Software/Rfam). The conserved miRNAs were subsequently annotated via alignment with miRBase 21.0 (http://www.mirbase.org/index.shtml), allowing two mismatches. The remaining unknown sRNAs were analyzed to predict novel miRNAs using Mireap (http://sourceforge.net/projects/mirea p/). According to recently published criteria, novel miRNAs were screened [38]. The mapped sequences were employed to predict secondary structures with RNAfold software (http://www.tbi.univie.ac.at/*ivo/RNA).

### Alignment of sequences and phylogenetic analysis

Precursor and mature sequences of miRNAs from *Arabidopsis*, grape and poplar were obtained from miRBase, Release 21 (http://www.mirbase.org/index.shtml). ClustalW was used to generate multiple alignments of nucleotide acid sequences and MEGA5 was used to generate phylogenetic analyses using the ML method [39]. ML phylogenetic trees were produced with 1000 bootstrap replications. Multiple sequence alignments of mature miRNAs were employed to generate a graphical representation of the consensus pattern using the web-based tool WebLogo (http://weblogo.berkeley.edu/logo.cgi, accessed 25 Feb. 2015).

### Cloning hickory miRNA precursors

A modified CTAB method was used to extract total RNA from hickory tissues [40]. The total RNA from hickory was transcribed into cDNA using an M-MLV reverse transcriptase (RNase H-) kit (Takara, China). The cDNA was amplified via PCR using primer pairs designed based on predicted hickory miRNA precursors with Primer Premier 5.0. The PCR products were then cloned into the pMD19-T (simple) vector (Takara, China), and further sequencing was conducted at Sangon Biotech (Shanghai, China) (Additional file 1: Table S1).

### qRT-PCR of miRNAs and their target mRNAs

A comparative qRT-PCR expression analysis for different tissues (roots, stems, leaves, female and fruits) and also for different female floral development stages (A1, A2, A3, and A4) were performed. As previous described [41], miRNAs were chosen for stem-loop RT-PCR. Briefly, total RNA was hybridized with a miRNA-specific stem-loop primer. About 500 ng total RNA was reverse-transcribed into cDNA in a 10-μl reaction volume using M-MLV Reverse Transcriptase (D2639A, Takara). qRT-PCR was performed with Platinum® SYBR® Green qPCR SuperMix-UDG (Invitrogen, C11744500). The 20-μl reaction system included 1 μl of the RT product, 4 μl of each primer (5 μM), 10 μl of 2× SYBR Green reaction mix and 5 μl of ddH_2_O. All cDNA samples were assayed in triplicate. The primers employed for these assays are listed in Additional file 2: Table S2 and Additional file 2: Table S3. 5.8S rRNA was used as a reference gene. The fold changes of each gene were calculated using the 2^−△△Ct^ method [42].

### Statistics

Least-square means (LS means) and analysis of variance (ANOVA; GLM procedure) were performed using Microsoft Office Excel 2007.

## Additional files

Additional file 1: **Table S1.** Cloning of hickory miRNA precursors primers. **Table S2.** Real-Time PCR of hickory miRNA primers. **Table S3.** Real-Time PCR of hickory miRNA targets primers and target sequencses. **Table S4.** Hickory miRNA sequences. **Table S5.** Conserved and Novel different expression miRNAs identified in *Carya cathayensis*. (XLS 259 kb)

Additional file 2: **Figure S1.** Conserved and Novel miRNA nucleotide bias at each position. a, Represent conserved miRNA nucleotide bias. b, Represent novel miRNA nucleotide bias. **Figure S2.** Real-Time PCR analysis of miRNA expression. Real-time PCR was performed with different flower timing. Each bar represents an average of three independent reactions, including both biological and technical replicates. Error bars indicate SD. **Figure S3.** Real-Time PCR analysis of miRNA expression. Real-time PCR was performed with different tissues. Each bar represents an average of three independent reactions, including both biological and technical replicates. Error bars indicate SD. **Figure S4.** The maximum likelihood (ML) phylogenetic tree reconstruction using precursor miRNA family sequences from Arabidopsis (ath), grape (vvi), poplar (ptc), and hickort (cca). MUSCLE alignment and ML were used for tree generation. **Figure S5.** Sequence logo showing a consensus sequence generated from the multiple alignments of miRNA families from four different plant species. a, The miR160, miR162, miR168, miR319, miR390, miR398 and miR408 represent highly conserved miRNAs. b, The miR172 and miR397 represent moderately conserved miRNAs. c, The miR169 represent lowly conserved miRNAs. **Figure S6.** RT-PCR analysis of miRNA and its targets expression. RT-PCR was performed with different tissues. Each bar represents an average of three independent reactions, including both biological and technical replicates. Error bars indicate SD.(DOC 3078 kb)

## Abbreviations

AF: April Flower
*AGO*: Argonaute
*AP2*: Apetala2
*ARF*: Auxin response factors
*DCL1*: Dicer-Like1
EST: Sequenced tag
*HD-ZIPIII*: Class III homeodomain-leucine zipper
*LOM*: Lost meristems
MF: March Flower
miRNA: microRNA
ML: Maximum likelihood
*NF-YA1*: Nuclear Factor Y, Subunit A1
ORF: Open reading frame
qRT-PCR: Quantitative real-time PCR
RISC: RNA-induced silencing complex
*SBP*: Squamosa promoter-binding protein
sRNA: Small RNA
UTR: Untranslated regions
