# Supplementary material for: Genome-wide analysis of miRNAs in Carya cathayensis
Source: BMC Plant Biol. 2017 Nov 29;17:228. doi: 10.1186/s12870-017-1180-6 (PMC5708078; doi:10.1186/s12870-017-1180-6)
Supplement: Supplementary file 2 — Figure S1. Conserved and Novel miRNA nucleotide bias at each position. a, Represent conserved miRNA nucleotide bias. b, Represent novel miRNA nucleotide bias. Figure S2. Real-Time PCR analysis of miRNA expression. Real-time PCR was performed with different flower timing. Each bar represents an average of three independent reactions, including both biological and technical replicates. Error bars indicate SD. Figure S3. Real-Time PCR analysis of miRNA expression. Real-time PCR was performed with different tissues. Each bar represents an average of three independent reactions, including both biological and technical replicates. Error bars indicate SD. Figure S4. The maximum likelihood (ML) phylogenetic tree reconstruction using precursor miRNA family sequences from Arabidopsis (ath), grape (vvi), poplar (ptc), and hickort (cca). MUSCLE alignment and ML were used for tree generation. Figure S5. Sequence logo showing a consensus sequence generated from the multiple alignments of miRNA families from four different plant species. a, The miR160, miR162, miR168, miR319, miR390, miR398 and miR408 represent highly conserved miRNAs. b, The miR172 and miR397 represent moderately conserved miRNAs. c, The miR169 represent lowly conserved miRNAs. Figure S6. RT-PCR analysis of miRNA and its targets expression. RT-PCR was performed with different tissues. Each bar represents an average of three independent reactions, including both biological and technical replicates. Error bars indicate SD.(DOC 3078 kb) [file 12870_2017_1180_MOESM2_ESM.doc]

Genome-wide analysis of miRNAs in *Carya cathayensis*

Zhichao Sun^1^, Liangsheng Zhang^2^,*, & Zhengjia Wang^1^,*

^1^Nurturing Station for the State Key Laboratory of Subtropical Silviculture, School of Forestry and Biotechnology, Zhejiang Agriculture and Forestry University, Hangzhou 311300, China

^2^Center for Genomics and Biotechnology, Haixia Institute of Science and Technology, Fujian Agriculture and Forestry University, Fuzhou 350002, China

*Corresponding author: Zhengjia Wang and Liangsheng Zhang

School of Forestry and Biotechnology, Zhejiang A and F University, Dong Hu Campus, 88 Northern Circle Road, Linan 311300, China

E-mail: [wzhj21@163.com](mailto:wzhj21@163.com); [fafuzhang@163.com](mailto:fafuzhang@163.com)

Fax: 0086(0)571 63732738

Tel: 0086(0)571 63743856

**Figures**


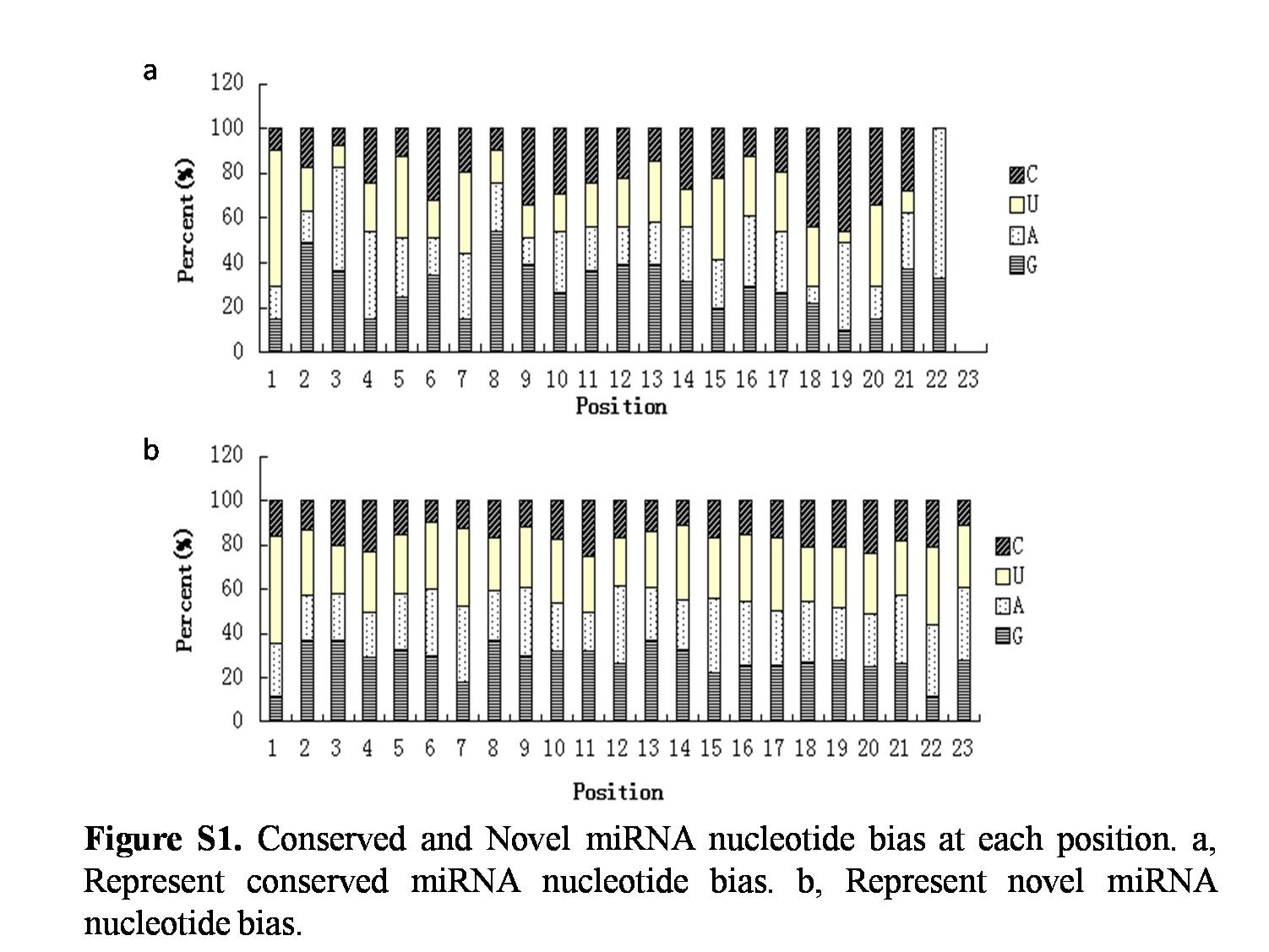


**Fig. S1** Conserved and novel miRNA nucleotide bias at each position. a, represent conserved miRNA nucleotide bias. b, represent novel miRNA nucleotide bias.


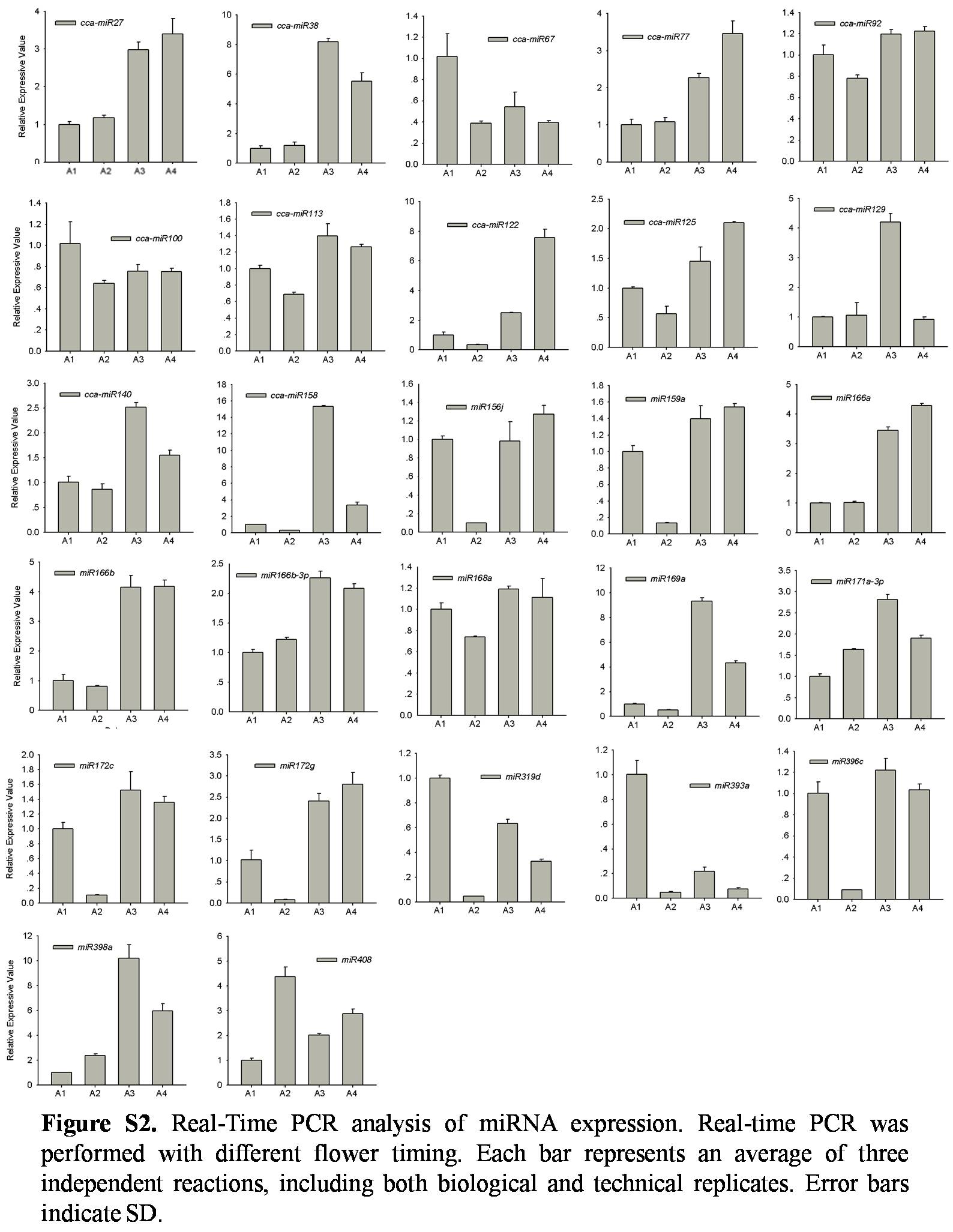


**Fig. S2** RT-PCR analysis of miRNA expression. RT-PCR was performed with different flower timing. Each bar represents an average of three independent reactions, including both biological and technical replicates. Error bars indicate SD.


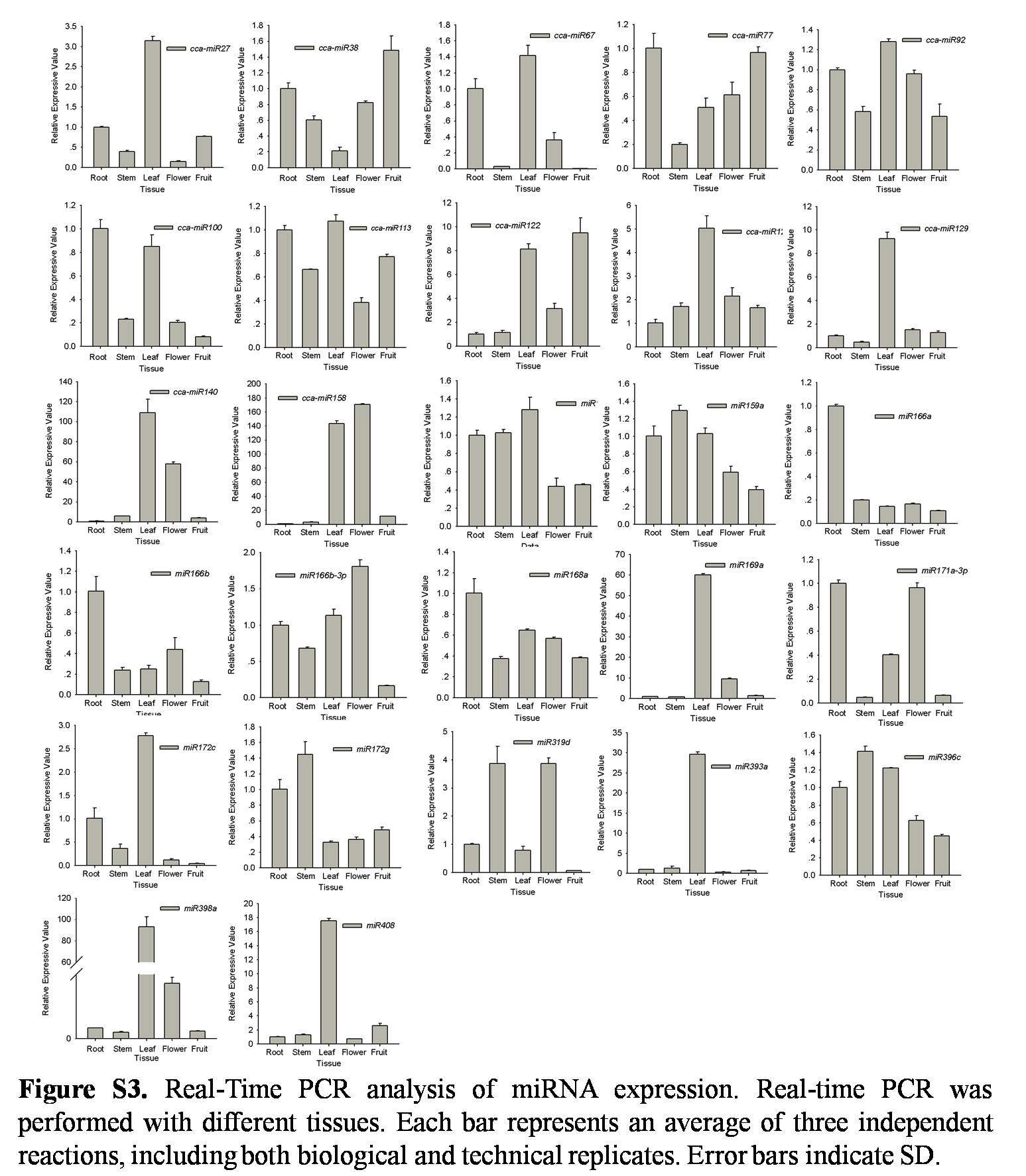


**Fig. S3** RT-PCR analysis of miRNA expression. RT-PCR was performed with different tissues. Each bar represents an average of three independent reactions, including both biological and technical replicates. Error bars indicate SD.


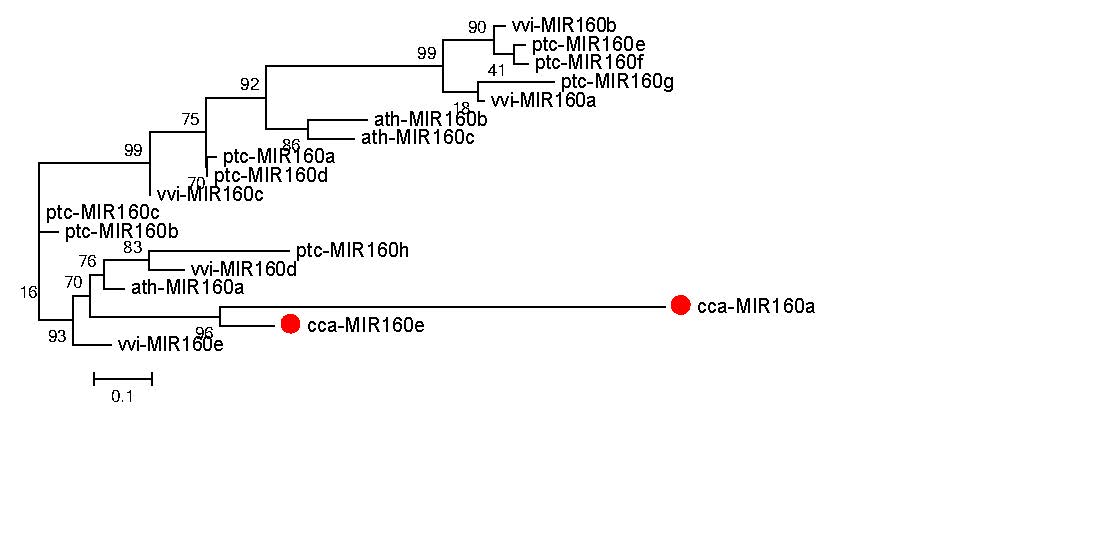


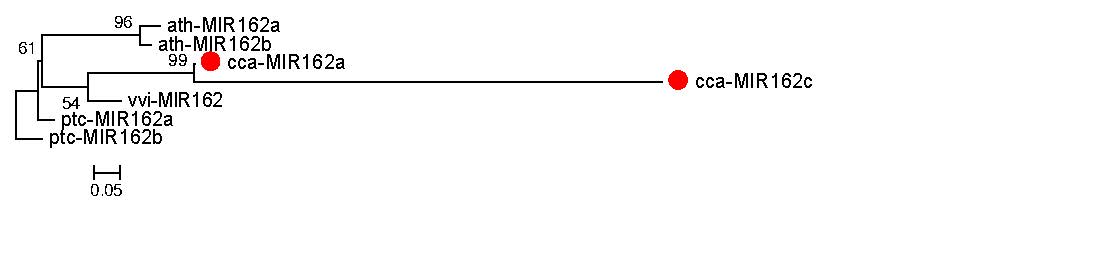


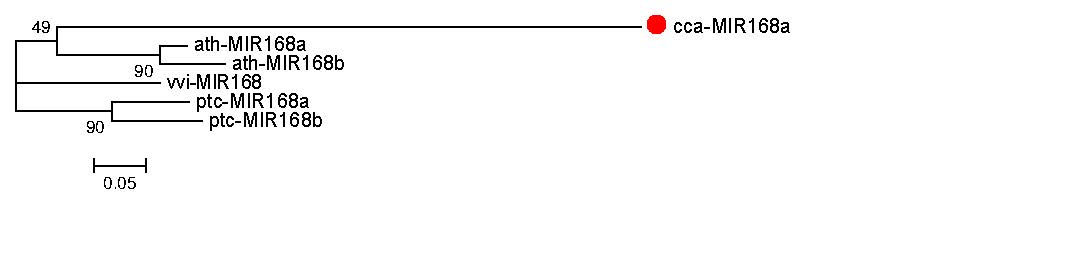


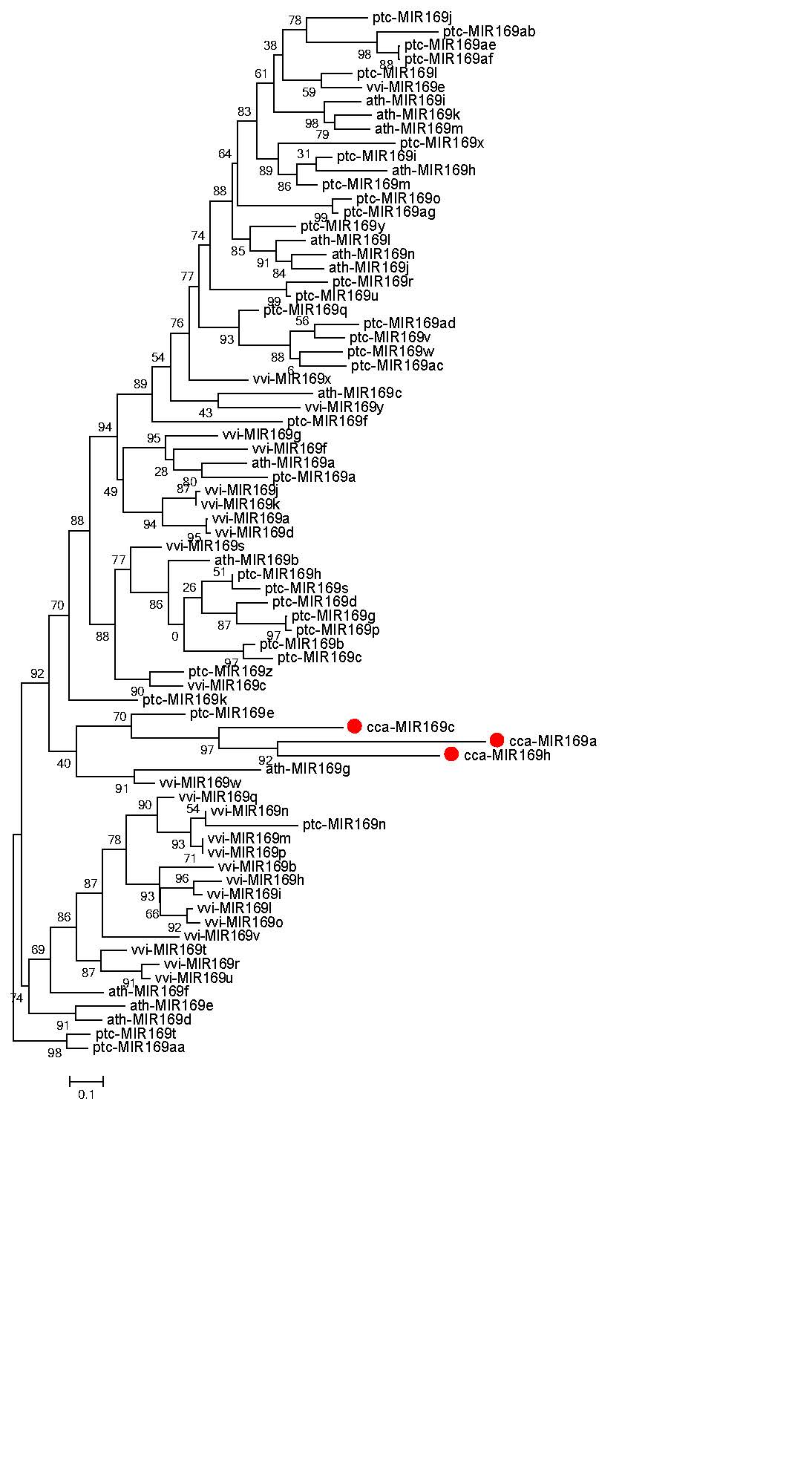


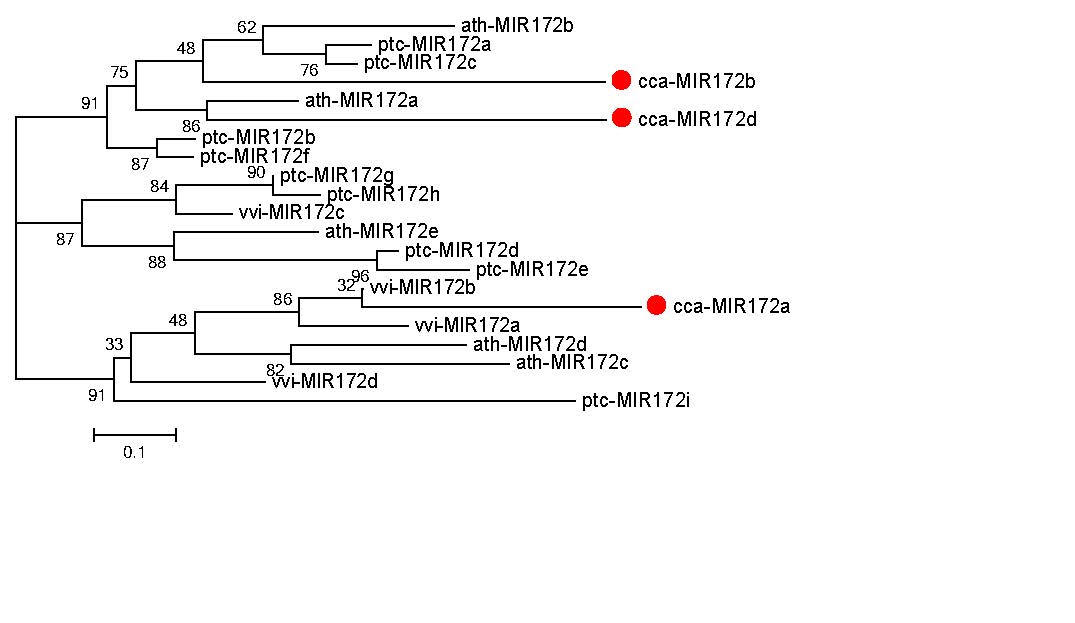


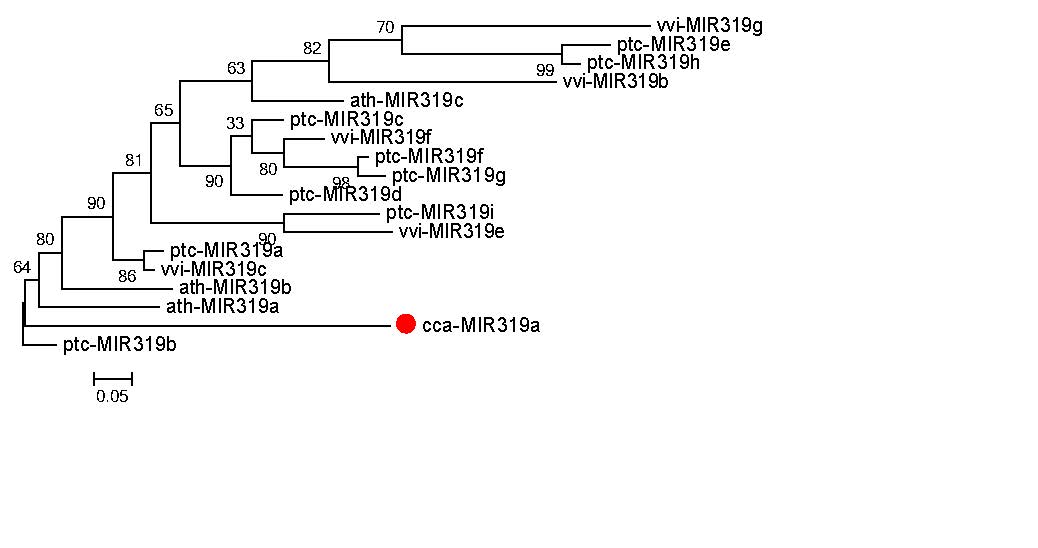


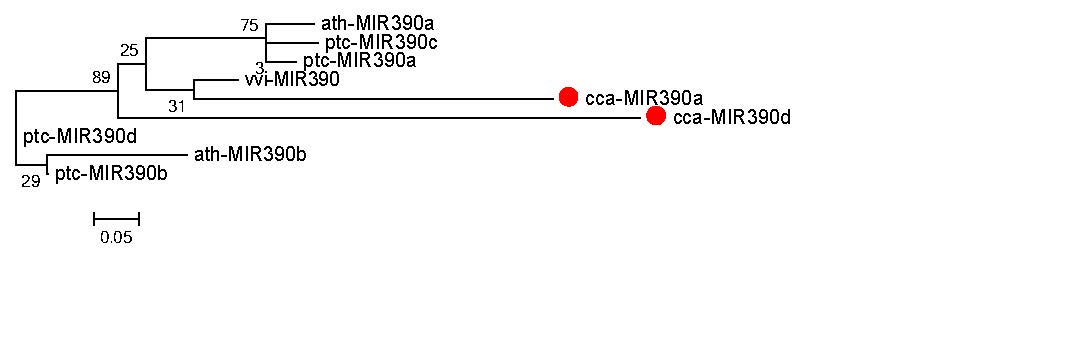


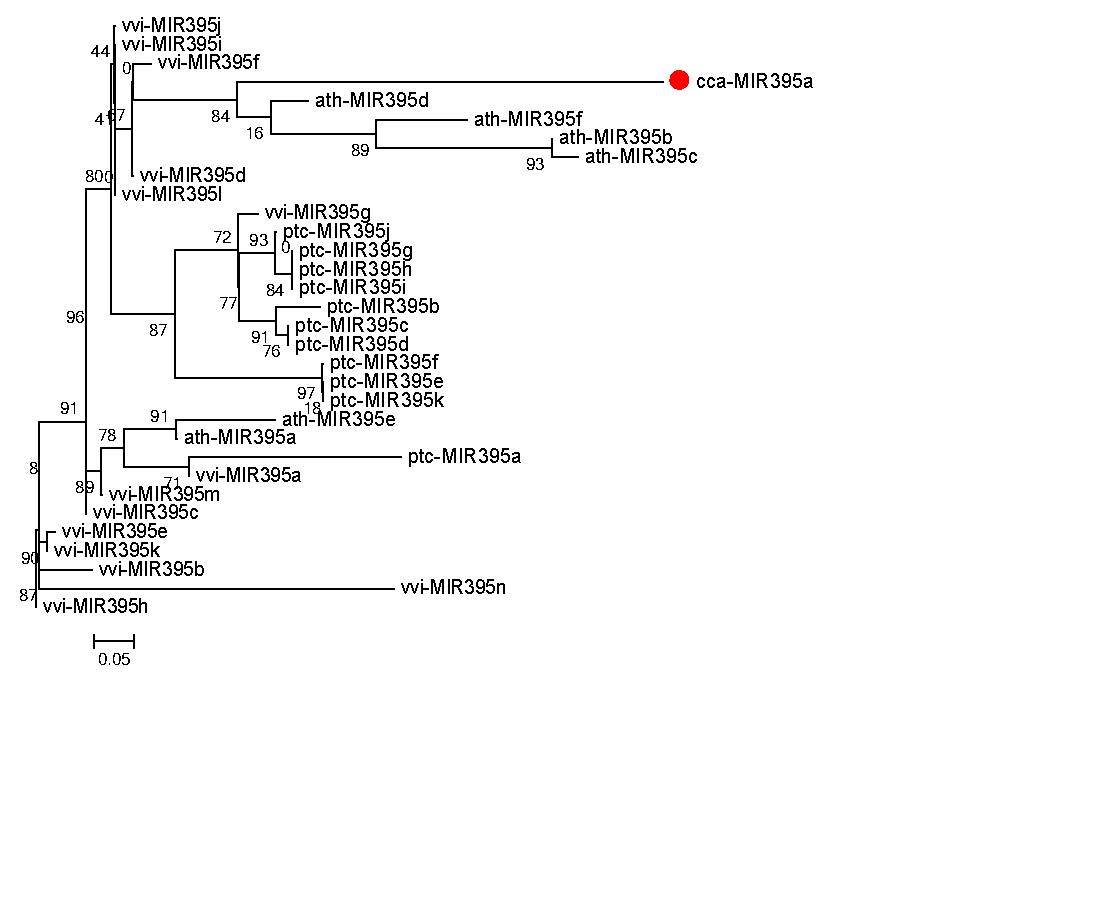


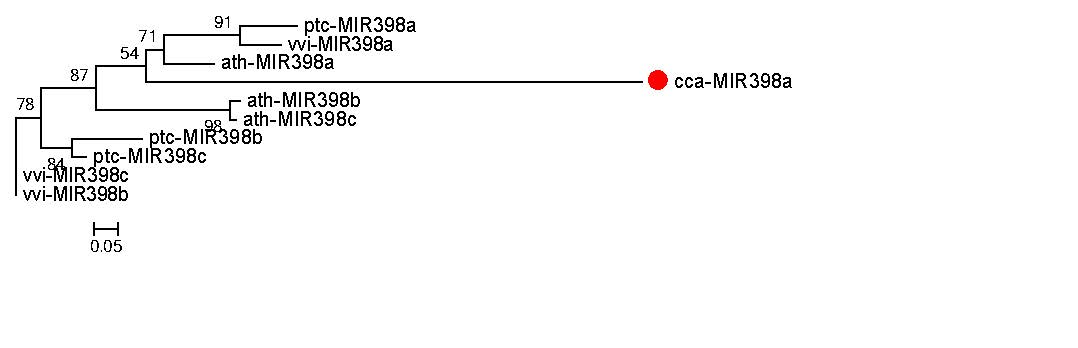


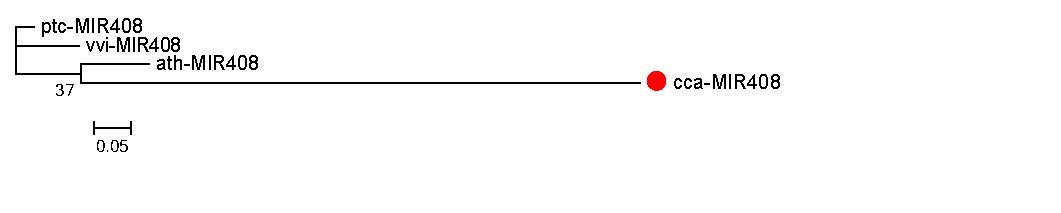


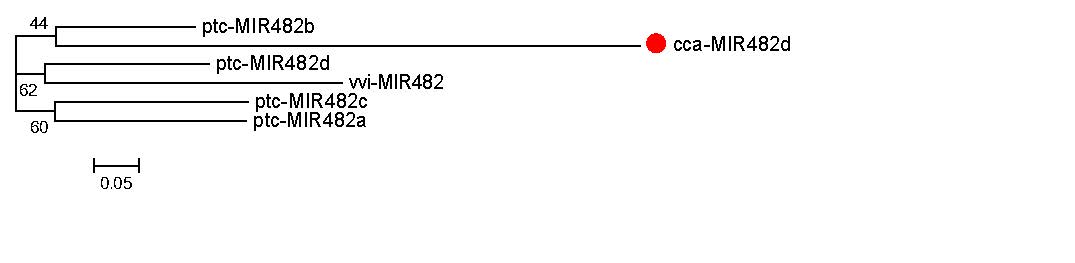


**Figure S4.** The maximum likelihood (ML) phylogenetic tree reconstruction using precursor miRNA family sequences from Arabidopsis (ath), grape (vvi), poplar (ptc), and hickort (cca). MUSCLE alignment and ML were used for tree generation. The miRBase accession numbers as follows: ath-MIR160a (MI0000190), ath-MIR160b (MI0000191), ath-MIR160c (MI0000192), ptc-MIR160a (MI0002201), ptc-MIR160b (MI0002202), ptc-MIR160c (MI0002203), ptc-MIR160d (MI0002204), ptc-MIR160e (MI0002205), ptc-MIR160f (MI0002206), ptc-MIR160g (MI0002207), ptc-MIR160h (MI0002208), vvi-MIR160a (MI0006496), vvi-MIR160b (MI0006497), vvi-MIR160c (MI0006498), vvi-MIR160d (MI0006499), vvi-MIR160e (MI0006501), ath-MIR162a (MI0000194), ath-MIR162b (MI0000195), ptc-MIR162a (MI0002209), ptc-MIR162b (MI0002210), vvi-MIR162 (MI0006502), ath-MIR168a (MI0000210), ath-MIR168b (MI0000211), ptc-MIR168a (MI0002243), ptc-MIR168b (MI0002244), vvi-MIR168 (MI0006520), ath-MIR172a (MI0000215), ath-MIR172b (MI0000216), ath-MIR172c (MI0000991), ath-MIR172d (MI0000992), ath-MIR172e (MI0001089), ptc-MIR172a (MI0002287), ptc-MIR172b (MI0002288), ptc-MIR172c (MI0002289), ptc-MIR172d (MI0002290), ptc-MIR172e (MI0002291), ptc-MIR172f (MI0002292), ptc-MIR172g (MI0002293), ptc-MIR172h (MI0002294), ptc-MIR172i (MI0002295), vvi-MIR172a (MI0006544), vvi-MIR172b (MI0006545), vvi-MIR172c (MI0006546), vvi-MIR172d (MI0006547), ath-MIR319a (MI0000544), ath-MIR319b (MI0000545), ath-MIR319c (MI0001086), ptc-MIR319a (MI0002296), ptc-MIR319b (MI0002297), ptc-MIR319c (MI0002298), ptc-MIR319d (MI0002299), ptc-MIR319e (MI0002300), ptc-MIR319f (MI0002301), ptc-MIR319g (MI0002302), ptc-MIR319h (MI0002303), ptc-MIR319i (MI0002304), vvi-MIR319b (MI0006548), vvi-MIR319c (MI0006549), vvi-MIR319e (MI0007951), vvi-MIR319f (MI0006550), vvi-MIR319g (MI0006551), ath-MIR390a (MI0001000), ath-MIR390b (MI0001001), ptc-MIR390a (MI0002305), ptc-MIR390b (MI0002306), ptc-MIR390c (MI0002307), ptc-MIR390d (MI0002308), vvi-MIR390 (MI0006552), ath-MIR395a (MI0001007), ath-MIR395b (MI0001008), ath-MIR395c (MI0001009), ath-MIR395d (MI0001010), ath-MIR395e (MI0001011), ath-MIR395f (MI0001012), ptc-MIR395a (MI0002315), ptc-MIR395b (MI0002316), ptc-MIR395c (MI0002317), ptc-MIR395d (MI0002318), ptc-MIR395e (MI0002319), ptc-MIR395f (MI0002320), ptc-MIR395g (MI0002321), ptc-MIR395h (MI0002322), ptc-MIR395i (MI0002323), ptc-MIR395j (MI0002324), ptc-MIR395k (MI0022045), vvi-MIR395a (MI0006556), vvi-MIR395b (MI0006557), vvi-MIR395c (MI0006558), vvi-MIR395d (MI0006559), vvi-MIR395e (MI0006560), vvi-MIR395f (MI0006561), vvi-MIR395g (MI0006562), vvi-MIR395h (MI0006563), vvi-MIR395i (MI0006564), vvi-MIR395j (MI0006565), vvi-MIR395k (MI0006566), vvi-MIR395l (MI0006567), vvi-MIR395m (MI0006568), vvi-MIR395n (MI0007954), ath-MIR398a (MI0001017), ath-MIR398b (MI0001018), ath-MIR398c (MI0001019), ptc-MIR398a (MI0002335), ptc-MIR398b (MI0002336), ptc-MIR398c (MI0002337), vvi-MIR398a (MI0006572), vvi-MIR398b (MI0007958), vvi-MIR398c (MI0007959), ath-MIR408 (MI0001080), ptc-MIR408 (MI0002352), vvi-MIR408 (MI0006578), ptc-MIR482a (MI0002397), ptc-MIR482b (MI0022043), ptc-MIR482c (MI0022048), ptc-MIR482d (MI0022007 ), vvi-MIR482 (MI0007971).


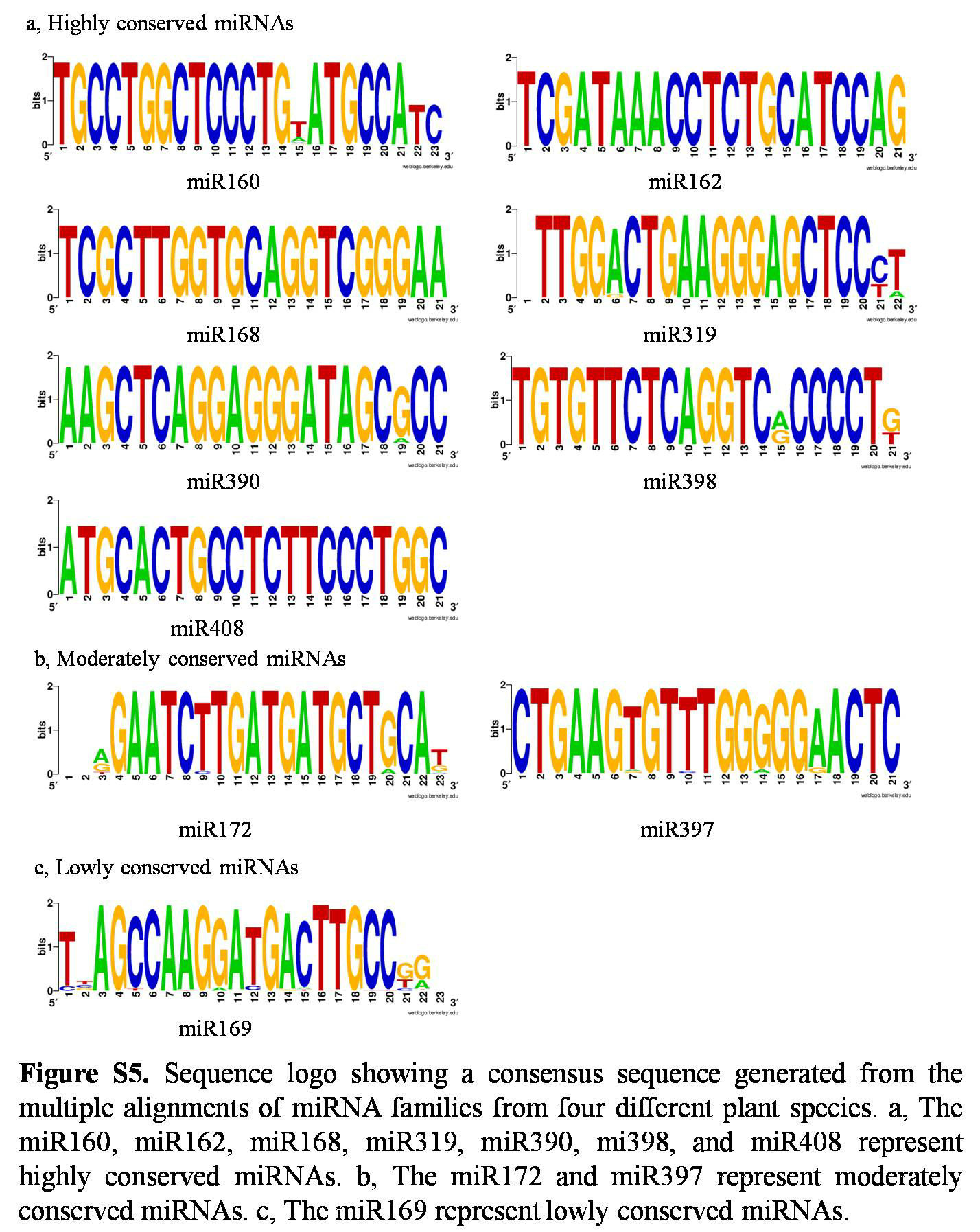


**Fig. S5** Sequence logo showing a consensus sequence generated from the multiple alignments of miRNA families from four different plant species. a, The miR160, miR162, miR168, miR319, miR390, miR398 and miR408 represent highly conserved miRNAs. b, The miR172 and miR397 represent moderately conserved miRNAs. c, The miR169 represent lowly conserved miRNAs.


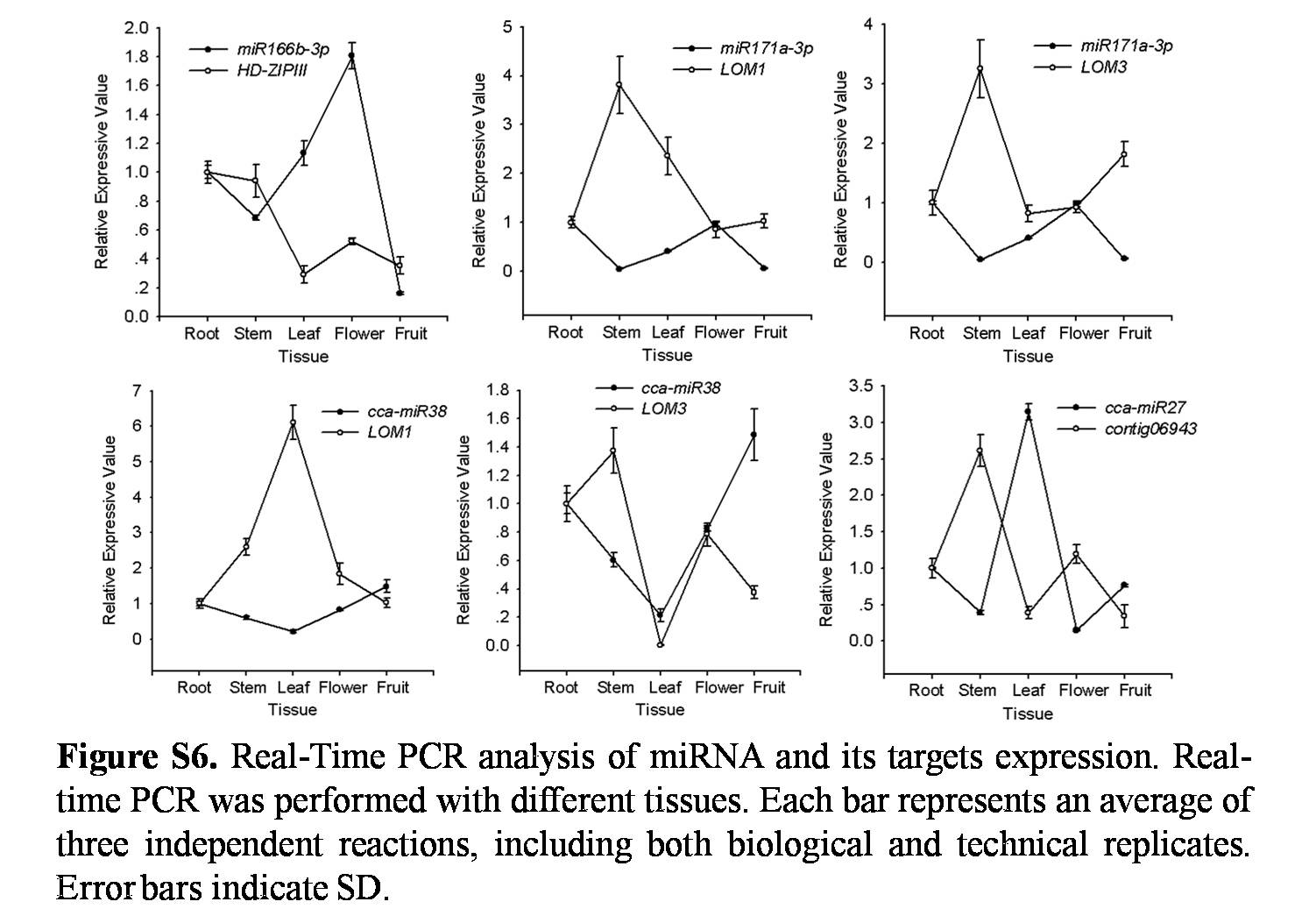


**Fig. S6** RT-PCR analysis of miRNA and its targets expression. RT-PCR was performed with different tissues. Each bar represents an average of three independent reactions, including both biological and technical replicates. Error bars indicate SD.
